# *Mecp2* Deletion from Cholinergic Neurons Selectively Impairs Recognition Memory and Disrupts Cholinergic Modulation of the Perirhinal Cortex

**DOI:** 10.1523/ENEURO.0134-19.2019

**Published:** 2019-10-30

**Authors:** Elizabeth C. Ballinger, Christian P. Schaaf, Akash J. Patel, Antonia de Maio, Huifang Tao, David A. Talmage, Huda Y. Zoghbi, Lorna W. Role

**Affiliations:** 1 Department of Neurobiology and Behavior, Stony Brook University, Stony Brook, New York 11794; 2 Program in Neuroscience, Stony Brook University, Stony Brook, New York 11794; 3 Medical Scientist Training Program, Stony Brook University, Stony Brook, New York 11794; 4 Center for Nervous System Disorders, Stony Brook University, Stony Brook, New York 11794; 5 Department of Pharmacological Sciences, Stony Brook University, Stony Brook, New York 11794; 6 Program in Developmental Biology, Baylor College of Medicine, Houston, Texas 77030; 7 Jan and Dan Duncan Neurological Research Institute at Texas Children’s Hospital, Baylor College of Medicine, Houston, Texas 77030; 8 Department of Molecular and Human Genetics, Baylor College of Medicine, Houston, Texas 77030; 9 Department of Neurosurgery, Baylor College of Medicine, Houston, Texas 77030; 10 Howard Hughes Medical Institute, Baylor College of Medicine, Houston, Texas 77030; 11 Institute of Human Genetics, Heidelberg University, 69120 Heidelberg, Germany

**Keywords:** acetylcholine, Mecp2, perirhinal, recognition, Rett Syndrome

## Abstract

Rett Syndrome is a neurological disorder caused by mutations in the gene encoding methyl CpG binding protein 2 (MeCP2) and characterized by severe intellectual disability. The cholinergic system is a critical modulator of cognitive ability and is affected in patients with Rett Syndrome. To better understand the importance of MeCP2 function in cholinergic neurons, we studied the effect of selective *Mecp2* deletion from cholinergic neurons in mice. Mice with *Mecp2* deletion from cholinergic neurons were selectively impaired in assays of recognition memory, a cognitive task largely mediated by the perirhinal cortex (PRH). Deletion of *Mecp2* from cholinergic neurons resulted in profound alterations in baseline firing of L5/6 neurons and eliminated the responses of these neurons to optogenetic stimulation of cholinergic input to PRH. Both the behavioral and the electrophysiological deficits of cholinergic *Mecp2* deletion were rescued by inhibiting ACh breakdown with donepezil treatment.

## Significance Statement

Rett Syndrome, a developmental disorder characterized by multiple deficits including intellectual disability, is caused by mutations in the *MECP2* gene. In this study, *Mecp2* was selectively deleted from cholinergic neurons in mice causing a specific impairment of recognition memory that was reversed following chronic administration of the acetylcholinesterase inhibitor donepezil. As recognition memory engages the perirhinal cortex, we examined the effects of *Mecp2* deletion from cholinergic neurons on the physiology of perirhinal cortical neurons and found a reduction in the variability of baseline firing and impaired responsiveness to optogenetic stimulation of cholinergic input. Our findings are consistent with a loss of encoding capacity in the perirhinal cortex and suggest a possible electrophysiological substrate for the altered profile of recognition memory performance.

## Introduction

Rett Syndrome is a childhood neurologic disorder that affects 1 in 10,000 girls and is caused by mutations in a gene known as *MECP2* (Rett, 1966; Hagberg et al., 1983; Lewis et al., 1992; Amir et al., 1999; Neul et al., 2010). The phenotype is complex and includes intellectual disability, breathing disturbances when awake, seizures, autonomic dysfunction, autistic features, stereotypies, locomotor defects, and gastrointestinal dysfunction. *MECP2* encodes methyl CpG binding protein 2, a transcriptional regulator whose loss leads to both decreases and increases in gene expression (Chahrour et al., 2008). Discerning what cell types and *Mecp2* target genes contribute to which aspects of the Rett Syndrome phenotype has posed a significant challenge. This challenge has been addressed by selectively removing *Mecp2* from distinct neuronal populations, revealing which neuronal populations and brain regions contribute to the key features of the disorder. Genetically targeted approaches have previously been applied to the GABAergic, glutamatergic, Sim1-expressing, and aminergic systems; and have provided an added level of resolution in the investigation of the significance of these neurotransmitter systems to Rett Syndrome (Fyffe et al., 2008; Samaco et al., 2009; Chao et al., 2010; Meng et al., 2016).

Given that a key characteristic of individuals with Rett Syndrome is severe cognitive impairment (Rett, 1966; Hagberg et al., 1983; Neul et al., 2010) and the well established role of the cholinergic system in cognitive functions (for review, see Ballinger et al., 2016), we have used the *Mecp2* deletion approach to assess the role of MeCP2 in cholinergic neurons and the resulting phenotypes due to its loss. Prior studies in both humans and animal models have implicated alterations in acetylcholine (ACh) signaling in Rett Syndrome. Postmortem immunohistochemical studies of the brain from individuals with Rett Syndrome have shown profound cholinergic deficits: there are reduced numbers of choline acetyltransferase (ChAT)-positive cells in the basal forebrain, reduced ChAT and VAChT activity, and reduced cholinergic receptor expression (Kitt et al., 1990; Wenk and Mobley, 1996; Wenk, 1997; Wenk and Hauss-Wegrzyniak, 1999; Yasui et al., 2011). Likewise, mice lacking MeCP2 have shown reductions in both ACh and ChAT, dramatically attenuated cholinergic currents in electrophysiological experiments, and altered cholinergic receptor expression profiles (Ward et al., 2009; Ricceri et al., 2011; Oginsky et al., 2014; but see also Zhou et al., 2017). Finally, cholinergic marker reductions as evaluated by SPECT imaging *in vivo* have been correlated with clinical severity in patients with Rett Syndrome (Brašic´ et al., 2012).

To evaluate the potential contribution of the cholinergic system to phenotypes observed in Rett Syndrome in a systematic way, we have used a Cre-Lox system to selectively delete *Mecp2* from cholinergic neurons only (see Fig. 2*A*). We evaluated the performance of these mice on a number of cognitive tasks and found specific deficits in novel object recognition—behaviors that depend on intact functioning of the perirhinal cortex (PRH; for review, see Dere et al., 2007). We then explored the electrophysiological and molecular mechanisms underlying specific cognitive deficits in novel object recognition.

## Materials and Methods

### Animals

For electrophysiological, behavioral, and molecular experiments transgenic male mice expressing Cre recombinase under control of the *Chat* promoter (*Chat*-Cre; stock #006410, The Jackson Laboratory; RRID:IMSR_JAX:006410) maintained on a C57 background were crossed with female mice heterozygous for a floxed *Mecp2* allele (*Mecp2* flox; stock #007177, The Jackson Laboratory; RRID:IMSR_JAX:007177) maintained on a 129 background. This cross generated the following four different genotypes of male offspring: mice with no transgenes, mice with the *Chat*-Cre transgene only, mice with the *Mecp2* flox allele only, and mice with both transgenes (*Chat-Mecp2^-/y^*; see Fig. 2*A*). Male mice from the F1 generations of the original cross were used for experiments. Mice of all genotypes were born at the expected Mendelian ratios and were healthy appearing at birth. However, *Chat-Mecp2^-/y^*mice did exhibit a phenotype of reduced survival, with most *Chat-Mecp2^-/y^*mice dying between 16 and 36 weeks of age while all other genotypes lived for 40+ weeks. *Chat-Mecp2^-/y^*mice gained weight at rates similar to those of genetic controls and were generally healthy appearing until death, which was an acute/subacute event of unknown cause.

All mice were maintained on a 12 h light/dark cycle and allowed food and water *ad libitum*. Mice were either pair or group housed. No singly housed mice were used for behavioral experiments. The same cohort of mice was examined on both the partition test and the novel object recognition test. A separate cohort of mice was examined on conditioned fear testing. A subset of this second cohort also underwent Morris water maze testing. A third cohort of mice was used for electrophysiological experiments.

### Context-conditioned fear and cue-conditioned fear

Fear conditioning was performed as previously described (Takeuchi et al., 2011). This test was conducted when mice were 21 weeks old. Each mouse was placed in a sound-attenuated chamber and allowed to explore freely for 2 min. An 80 dB white noise, the conditioned stimulus (CS), was presented for 30 s; this was followed by a mild (2 s, 1 mA) footshock, the unconditioned stimulus (US). Two more CS–US pairings were presented with 2 min interstimulus intervals (ISIs). Context testing was conducted 1 d after conditioning in the same chamber. Cued testing with altered context was conducted on the same day, following the context testing, using a triangular box made of white opaque Plexiglas and vanilla scent presented behind the separation to change olfactory stimulus. Data acquisition, control of stimuli (i.e., tones and shocks), and data analysis were performed automatically using the Actimetrics FreezeFrame3 System (Coulbourn Instruments; RRID:SCR_014429). For context-conditioned fear, the percentage of time spent freezing in the conditioned context on testing day was calculated and compared between groups. For cue-conditioned fear, on testing day the cue-specific time spent freezing was calculated as follows: cue-specific freezing = (% time freezing during cue) − (% time freezing before cue). Cue-specific freezing was then compared between groups.

### Morris water maze

Morris water maze was performed as previously described (Takeuchi et al., 2011). This test was conducted when mice were 17 weeks old. A circular pool (120 cm in diameter) was filled with water (21 ± 1°C), in which nontoxic white tempera paint was mixed to make the surface opaque. For the invisible platform test, a white-colored platform was placed at the center in one of four quadrants of the pool (southwest area) and submerged 1 cm below the water surface so that it was invisible at water level. The location of the platform was fixed at the same quadrant, while the start position of swimming was varied. Mice were given eight trials per day (two blocks of four trials each) for 4 consecutive days, during which they were allowed 60 s to find the platform. Each trial was separated by an intertrial interval of 1–2 min, with each block separated by an interblock interval of at least 1 h. Once the mouse located the platform, it was permitted to stay on it for 10 s. If the mouse did not find the platform within 60 s, it was guided to the platform and placed on it for 20 s. To evaluate the spatial reference memory, all mice were given a probe trial following the training, at least 1 h after their last training trial on day 4. The probe trial consisted of removing the platform from the pool and allowing the mice to swim for 60 s in their search. A record was kept of the swimming time (in seconds) in the pool quadrant where the platform had previously been placed. During the visible platform test, a colored platform was placed in the quadrant 1 cm above the surface of the water, and its location was always varied randomly in each trial. Swim speed (in centimeters per second), latency time to find the platform (in seconds), and the time that each mouse swam in the target quadrant were recorded by video camera and analyzed by a computer-controlled video-tracking system (Ethovision XT, Noldus Information Technology; RRID:SCR_000441).

### Partition test

The partition test of social interaction was conducted when mice were 14 weeks old. Test subjects were individually housed in a standard cage divided by a perforated partition, as described previously (Moretti et al., 2005). Male partner mice of the same age were placed into the side opposite the test subject at least 18 h before testing social interaction. Recording of social interest (i.e., the amount of time test subjects spent at the partition actively interested in partner mice) was performed as described previously (Moretti et al., 2005).

### Recognition memory training

Recognition memory testing was conducted when mice were 20 weeks old. The behavioral apparatus consisted of two empty rodent cages cleaned with 40–50% EtOH, as follows: one cage was used as a habituation arena, and the other cage was used as the test arena. On each day for 5 consecutive days, mice were placed in the habituation arena for 5 min and then transferred to the test arena for 5 min. On days 1, 2, and 3, the test arena contained the same pair of two identical objects (Lego objects, ∼12 cm high). On day 4, one of the objects was replaced with a novel object (Lego object of same height, but of different color pattern and shape than the familiar object). On day 5, the test arena again contained the pair of identical familiar objects. The amount of time spent exploring each object was quantified during each session. The following behaviors were considered “exploration”: whisking the object, biting the object, touching the object, and nose oriented toward and within 2 cm of the object.

### Viral injection

Before the electrophysiological recording, a subset of mice underwent viral injection to facilitate optogenetic stimulation of cholinergic neurons. To target cholinergic neurons, we used a Cre-dependent virus, and the experiments were limited to *Chat*-Cre and *Chat-Mecp2^-/y^* mice. Mice for optogenetic experiments were anesthetized with isoflurane at 11 weeks of age and mounted on a stereotaxic frame (Kopf Instruments) with a heated stage. An incision in the scalp was made, and a small hole was drilled in the skull above the left nucleus basalis magnocellularis [NBM; coordinates from bregma: anteroposterior (AP), −0.7 mm; mediolateral (ML), 1.7 mm; *z*-axis, −4.0 mm]. A total of 0.5 μl of either AAV9-Ef1a-DIO-ChETA-eYFP or AAV9-CAG-DIO-oChIEF-tdTomato was injected using a microsyringe (Hamilton). Mice were used for electrophysiological recording 3 weeks after infection.

### Electrophysiological recording

For electrophysiological experiments, mice that were at least 13 weeks of age were anesthetized with isoflurane and placed on a surgical stereotax (Kopf Instruments) with a heated stage. A craniotomy over the left perirhinal cortex was performed and a tungsten electrode of either 1 or 5 MΩ (A-M Systems) was positioned into the posterior PRH (coordinates from bregma: AP, −3.25 mm; *z*-axis, −3.35 to −3.85 mm; ML from temporal ridge, −200 to +500 μm). Extracellular recordings were preamplified by the head stage of A-M Systems amplifier. For optogenetic experiments, mice (i.e., *Chat* Cre mice with or without *Mecp2* flox) received an additional craniotomy over the left NBM through which a 1 MΩ parylene-C-insulated tungsten electrode (A-M Systems) and a 200 μm optical fiber (Thorlabs) coupled to a 473 nm laser (Shanghai Dream Lasers Technology) were positioned in the NBM.

Signals were acquired at a sampling rate of 40 kHz and bandpass filtered at 100–1000 Hz by the amplifier (A-M Systems) before being passed through a Humbug Noise Eliminator (A-M Systems) and then displayed on a Tektronix TDS 2014B oscilloscope and fed to a Cambridge Electronic Design 1401 data board for visualization and collection using Spike 2 software (Cambridge Electronic Design). Laser stimuli used for optical stimulation of cholinergic neurons consisted of 20 laser pulses of 1 ms duration delivered at a frequency of 10 Hz.

### Relocalization of recording site

At the end of each recording session, an electrolytic lesion was created by passing 100 μA of current for 45 s through the recording electrode to facilitate relocalization of the recording site. The mouse was then perfused transcardially and brain slices were obtained as discussed below.

For a subset of mice, slices containing the perirhinal cortex were stained using NeuroTrace (Thermo Fisher Scientific) blue fluorescent Nissl stain. The perirhinal cortex was defined histologically as per Beaudin et al. (2013). In short: the medial border was defined by the external capsule, the dorsal border was distinguished by the loss of the prominent layer IV seen in the dorsally adjacent temporal association cortex, the ventral border was distinguished by loss of the prominent layer II seen in the ventrally adjacent entorhinal cortex.

For all mice, slices containing the PRH were imaged on a stereoscope (Zeiss). For mice used for optogenetic experiments, slices containing the NBM were imaged to confirm viral expression.

### Sample preparation for light and confocal microscopy

Mice were anesthetized with a 9:1 mixture of ketamine and xylazine and transcardially perfused with 1× PBS followed by 4% PFA. Brains were removed and postfixed overnight in 4% PFA before being sucrose equilibrated and frozen in OCT (optimal cutting temperature) compound. Brains were then cryosectioned (Leica Biosystems) at 50 μm thickness.

### Immunohistochemistry

For Nissl staining brain slices were blocked and permeabilized for 30 min in 1× PBS with 5% donkey serum and 0.1% Triton X-100, and then incubated for 90 min in a 1:200 dilution (in blocking/permeabilization solution) of the NeuroTrace blue fluorescent Nissl stain (Thermo Fisher Scientific) followed by three 5 min washes in 1× PBS. Slices were mounted with DAPI Fluoromount-G (Southern Biotech). All steps were performed at room temperature.

For MeCP2 and ChAT staining, brain slices were blocked and permeabilized for 1 h at room temperature in 1× PBS with 5% donkey serum and 0.5% Triton X-100 and then incubated for 48 h on a shaker at 4°C in 1:200 rabbit anti-MeCP2 (catalog #3456, Cell Signaling Technology; RRID:AB_2143849) or 1:200 goat anti-ChAT (EMD Millipore; RRID:AB_2079751) diluted in blocking solution. Slices were then washed three times for 10 min each in 1× PBS and then incubated overnight in 1:500 Invitrogen goat anti-rabbit (Thermo Fisher Scientific) or 1:1000 Invitrogen donkey anti-goat (Thermo Fisher Scientific). After three 10 min washes in 1× PBS, slices were mounted with Vectashield (Vector Laboratories; RRID:AB_2336788) with DAPI and imaged on a confocal microscope.

### Electrophysiological data analysis

Extracellular recordings were sorted off-line using the Offline Sorter (Plexon). Features of the waveforms were extracted, and individual units were demarcated by manually identifying clusters of waveforms in a two-dimensional feature space of spike properties (Gray et al., 1995). The quality of each sort was rated according to the isolation distance between clusters within the recording. Only recordings of high sort quality, with <5% overlap with other clusters, were used for further analysis. Units with firing rates <0.05 Hz were excluded from further analysis.

The variability of the baseline firing rate was quantified by calculating the Fano factor (FF) of the firing rate computed for each 10 s bin during the 300 s preceding optical stimulation.

Responses to optogenetic stimulation were evaluated using a permutation test of the *F* statistic with 10,000 permutations comparing interspike intervals occurring within the 140 s immediately before and after stimulation. To identify delayed responders, the same analysis was performed comparing the 140 s immediately before stimulation and a sliding 140 s window following stimulation. Each slide step was 70 s. A response was detected if the resultant *p* value from these permutation tests was <0.05. A unit was considered to exhibit an “early laser” response if a response was detected in the first 140 s window following stimulation. A unit was considered to exhibit a “delayed” response if a response was detected at any later window within the first 560 s following stimulation. If no responses were detected within the first 560 s following stimulation, the unit was considered to have no response.

### Donepezil pump implantation

A subset of *Chat-Mecp2^-/y^* mice generated using the same breeding strategy as described above underwent subcutaneous implantation of an osmotic minipump (model 2006, Alzet). Mice were anesthetized with isoflurane, and an incision was created over the left shoulder or caudal skull. Hemostats were lubricated with saline and then passed through the incision and used to open the subcutaneous space by separating the skin from the subcutaneous fascia. The pump was then implanted in this space, and the mouse was allowed to recover for 2 weeks. Pumps delivered either sterile saline or donepezil HCl (Biotang) in saline at a dose of 0.3 mg/kg/d.

### Statistics

Performance on the partition test and Morris water maze was compared using a repeated-measures ANOVA design with genotype and testing session included as factors. *Post hoc* pairwise comparisons were performed using the Tukey’s test. Performance on conditioned fear tests, baseline firing rate, firing rate variability, and performance on the test day of Novel Object Recognition for non-drug-treated mice were compared using the Kruskal–Wallis test. Response rates to optogenetic stimulation were compared between genotypes using a χ^2^ test of homogeneity, the Cressie–Read Power-Divergence Statistic method, as this method is modestly superior to the traditional Pearson method for small tables (Rudas, 1986; Thorvaldsen et al., 2010). All other comparisons were performed with the Wilcoxon rank sum test. The repeated-measures ANOVA was performed in SPSS (IBM; RRID:SCR_002865). All other comparisons were performed in Matlab (MathWorks; RRID:SCR_001622). Details of the statistical analysis are summarized in Table 1.

**Table 1:** Statistical methods by figure

Set of data	Type of analysis	Results of analysis	
Figure 2			
*B*: time to reach platform in Morris water maze: group x testing session interaction	Two-way repeated-measures ANOVA	*F*_(9,100)_ = 0.902	*p =* 0.527
*C*: comparison by genotype of the percentage of time spent freezing to context	Kruskal–Wallis test	*H*_(3)_ = 4.64	*p* = 0.20
*D*: comparison by genotype of the percentage of time spent freezing to cue	Kruskal–Wallis test	*H*_(3)_ = 6.46	*p* = 0.09
*E*: comparison by genotype of time with novel/time with familiar on test day	Kruskal–Wallis test	*H*_(3)_ = 22.97	*p* < 0.0005
*F*: time spent at partition: group × testing session interaction	Two-way repeated-measures ANOVA	*F*_(6,118)_ = 4.908	*p* < 0.0005
Figure 3			
*C*: comparison by genotype of Fano factor of firing rate	Kruskal–Wallis test	*H*_(3)_ = 8.92	*p* = 0.03
*D*: comparison by genotype of firing rate	Kruskal–Wallis test	*H*_(3)_ = 6.62	*p* = 0.085
Figure 5			
*C*: within-unit comparison of variance of ISI prestimulation and poststimulation	Permutation test of *F* statistic		
*D*: comparison by genotype of response rates	χ^2^ test of homogeneity	CR(2) = 6.02	*p* = 0.049
Figure 6			
*A*: comparison by treatment type of time with novel/time with familiar on test day	Wilcoxon rank sum test	Rank sum = 120	*p* = 0.03
*B*: comparison by treatment type of firing rate variability	Wilcoxon rank sum test	Rank sum = 293	*p* = 0.087
*D*: within unit comparison of variance of ISI prestimulation and poststimulation	Permutation test of *F* statistic		
*E*: comparison by treatment type of response rates	χ^2^ test of homogeneity	CR(2) = 4.15	*p* = 0.126

## Results

### Cognitive phenotyping of *Chat-Mecp2^-/y^* mice

**Figure 1.**
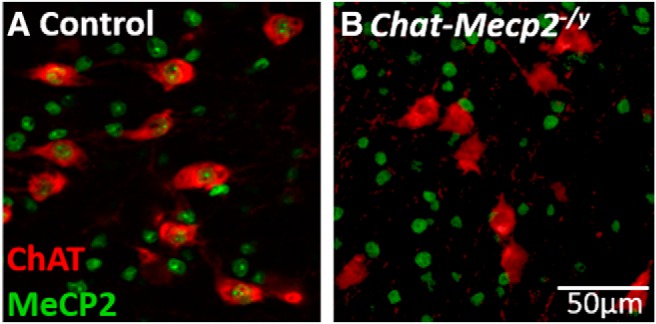
MeCP2 expression is eliminated in cholinergic neurons in *Chat-Mecp2^-/y^*mice. ***A***, In a representative slice from the basal forebrain of a no transgene control mouse, MeCP2 expression (green) is apparent in the nuclei of cholinergic neurons (anti-ChAT stained, red). ***B***, In a *Chat-Mecp2^-/y^*mouse, however, MeCP2 expression in cholinergic neurons was not detected.

**Figure 2.**
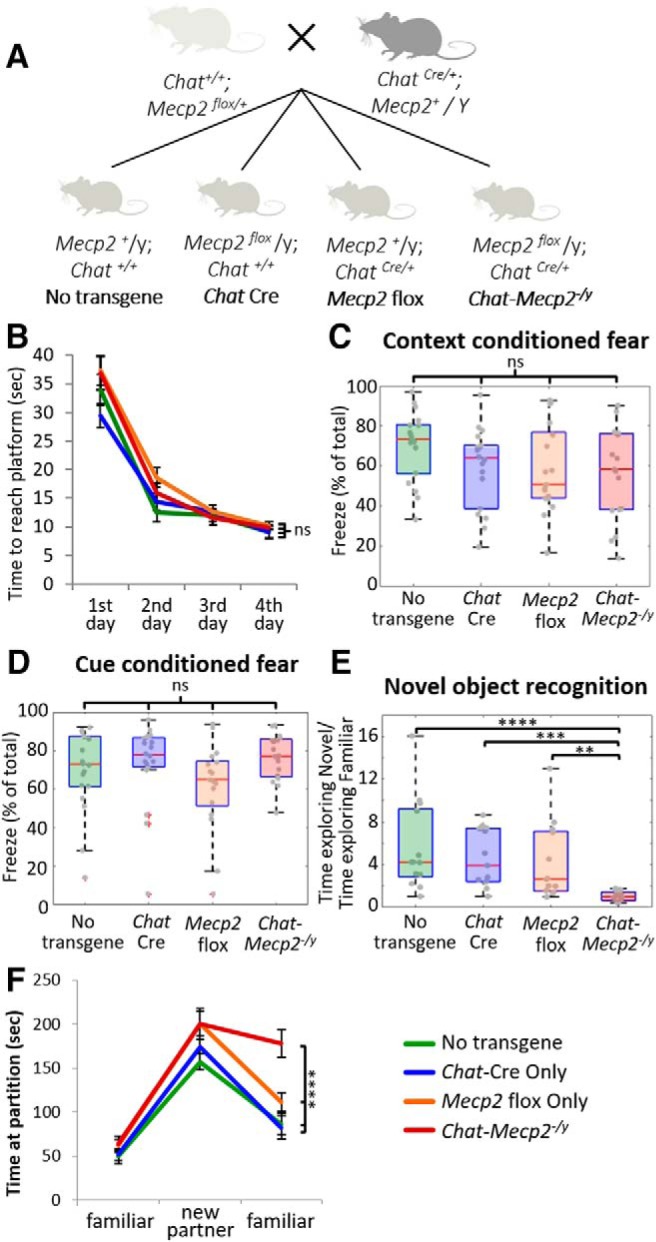
*Mecp2* cholinergic selective knock-out mice are impaired in recognition memory of either an object or a conspecific. ***A***, *Mecp2* flox mice were crossed with *Chat* Cre mice to generate *Mecp2* selective knock-out mice (*Chat-Mecp2^-/y^*) and all three genetic controls. ***A–E***, *Chat-Mecp2^-/y^* mice (***A***) performed at control levels on the Morris water maze (***B***; repeated-measures ANOVA: interaction effect for genotype × testing day: Wilks’ lambda = 0.827, *F*_(9,100)_ = 0.902, *p =* 0.527), context-conditioned fear (***C***; Kruskal–Wallis test: *H*_(3)_ = 4.64, *p* = 0.20), and cue-conditioned fear (***D***; Kruskal–Wallis test: *H*_(3)_ = 6.46, *p* = 0.09). ***E***, However, *Chat-Mecp2^-/y^* mice were impaired on novel object recognition (Kruskal–Wallis test: *H*_(3)_ = 22.97, *p <* 0.0005). *Post hoc* comparisons revealed that *Chat-Mecp2^-/y^* mice were significantly different from all three genetic controls (no transgene, *p* = 0.0001; *Chat* Cre, *p* = 0.0004; *Mecp2* flox, *p* = 0.0079). ***F***, *Chat-Mecp2^-/y^* mice showed reduced preference for the novel object introduced on day 4 (novel/familiar object ratio: mean, 1.038; SD, 0.48) than all three genetic controls (no transgene novel/familiar object ratio: mean, 5.6; SD, 4.30; *Chat* Cre novel/familiar object ratio: mean, 4.63; SD, 2.68; *Mecp2* flox novel/familiar object ratio: mean, 4.18; SD, 3.62). *Chat-Mecp2^-/y^* mice were also impaired on the partition test (repeated-measures ANOVA: interaction effect for genotype × behavior session: Wilks’ lambda = 0.64; *F*_(6,118)_ = 4.908; *p* < 0.0005). Pairwise comparisons revealed that *Chat-Mecp2^-/y^* were significantly different from both no transgene mice (*p* < 0.0005) and *Chat* Cre mice (*p* = 0.001), although the difference between *Chat-Mecp2^-/y^*and *Mecp2* flox mice did not reach significance (*p* = 0.079). On the partition test, *Chat-Mecp2^-/y^* mice were impaired in their ability to recognize a familiar mouse and spent longer interacting with the familiar mouse on re-presentation (mean, 178.05 s; SD, 63.02) than any of the genetic controls (no transgene: mean, 86.58 s; SD, 47.92; *Chat* Cre: mean, 82.38 s; SD, 51.89; *Mecp2* flox: mean, 111.31s; SD, 43.97). Error bars represent the SEM. **p ≤ 0.01, ***p ≤ 0.001, ****p ≤ 0.0005, ns = non significant.

As cholinergic signaling plays a vital role in mediating cognition (Ballinger et al., 2016) and intellectual disability is a central phenotype of *MECP2* disorders, we first asked whether selective *Mecp2* deletion from cholinergic neurons altered performance in several learning-related cognitive tasks. We used a Cre-lox system to selectively delete *Mecp2* from cholinergic neurons (Figs. 1, 2*A*). This system has previously been shown to effectively reduce Mecp2 expression, as measured by immunofluorescence (Ito-Ishida et al., 2015; Zhang et al., 2016).

*Chat-Mecp2^-/y^* mice performed at control levels on the Morris water maze, an assay of spatial memory (Fig. 2*B*; *n* = 12 for WT, *Chat* Cre, and *Mecp2* flox mice; *n* = 11 for *Chat-Mecp2^-/y^* mice; repeated-measures ANOVA: interaction effect for genotype × testing day: Wilks’ lambda = 0.827, *F*_(9,100)_ = 0.902, *p =* 0.527). Similarly, *Chat-Mecp2^-/y^* mice performed at control levels on the context-conditioned fear assay (Fig. 2*C*; *n* = 18 for WT and *Chat* Cre mice; *n* = 17 for *Mecp2* flox and *Chat-Mecp2^-/y^* mice; Kruskal–Wallis test, *H*_(3)_ = 4.64, *p* = 0.20) and cue-conditioned fear assay (Fig. 2*D*; *n* = 18 for WT and *Chat* Cre mice; *n* = 17 for *Mecp2* flox and *Chat-Mecp2^-/y^* mice; Kruskal–Wallis test, *H*_(3)_ = 6.46, *p* = 0.09).

*Chat-Mecp2^-/y^* mice were impaired in recognition memory, as measured by the novel object recognition task (Fig. 2*E*; *n* = 13 for no transgene, *Chat* Cre, and *Mecp2* flox mice; *n* = 12 for *Chat-Mecp2^-/y^* mice; Kruskal–Wallis test, *H*_(3)_ = 22.97, *p <* 0.0005). *Post hoc* comparisons revealed that *Chat-Mecp2^-/y^* mice showed significantly reduced preference for the novel object compared with all three genetic controls (no transgene, *p* = 0.0001; *Chat* Cre, *p* = 0.0004; *Mecp2* flox; *p* = 0.0079; no transgene novel/familiar object ratio: mean, 5.6; SD, 4.30; *Chat* Cre novel/familiar object ratio: mean, 4.63; SD, 2.68; *Mecp2* flox novel/familiar object ratio: mean, 4.18; SD, 3.62). No other significant differences were found. *Chat-Mecp2^-/y^* mice also performed abnormally in the partition test assay of social interaction and memory (Fig. 2*F*; *n* = 16/group; repeated-measures ANOVA: interaction effect for genotype × behavior session, Wilks’ lambda = 0.64, *F*_(6,118)_ = 4.908, *p* < 0.0005). Although *Chat-Mecp2^-/y^* mice originally interacted with the familiar mouse and the novel mouse at control levels, when the familiar mouse was reintroduced at the end of the trial, *Chat-Mecp2^-/y^* mice spent more time interacting with the familiar mice (mean, 178.05 s; SD, 63.02) than the control mice did (no transgene: mean, 86.58 s; SD, 47.92; *Chat* Cre: mean, 82.38 s; SD, 51.89; *Mecp2* flox: mean, 111.31 s; SD, 43.97). These data are consistent with the idea that selective deletion of *Mecp2* from cholinergic neurons alters the ability of the animals to distinguish between novel and familiar stimuli but did not affect social interactions per se. Testing on a comprehensive behavioral battery revealed no other behavioral deficits (Table 2).

**Table 2:** *Chat-Mecp2^-/y^* Mice have reduced survival but are not impaired on other behavioral phenotypes

Test	Phenotype tested	Phenotype present in *Chat-Mecp2^-/y^* mice ?
General health exam	Weight	NS from no transgene; *Mecp2* flox
General health exam	Stereotypies	No
Elevated plus maze	Anxiety-like behavior	No
Light/dark box	Anxiety-like behavior	No
Open field arena	Anxiety-like behavior	No
Open field arena	Hypo/hyperactivity	No
Rotarod	Motor learning	NS from no transgene; *Mecp2* flox
Rotarod	Motor coordination	NS from no transgene; *Mecp2* flox
Grip strength meter	Motor strength	No
Prepulse inhibition	Sensory gating	No
Passive avoidance	Contextual learning	No
Aging of animal	Reduced survival	Yes

### Electrophysiological recording of the PRH

Recognition memory is thought to engage circuits including the PRH, which receives cholinergic projections from neurons in the basal forebrain. Given that *Chat-Mecp2^-/y^* mice are selectively impaired in assays of recognition memory, we next asked whether there were any overt changes in electrophysiological profile of PRH neurons.

**Figure 3.**
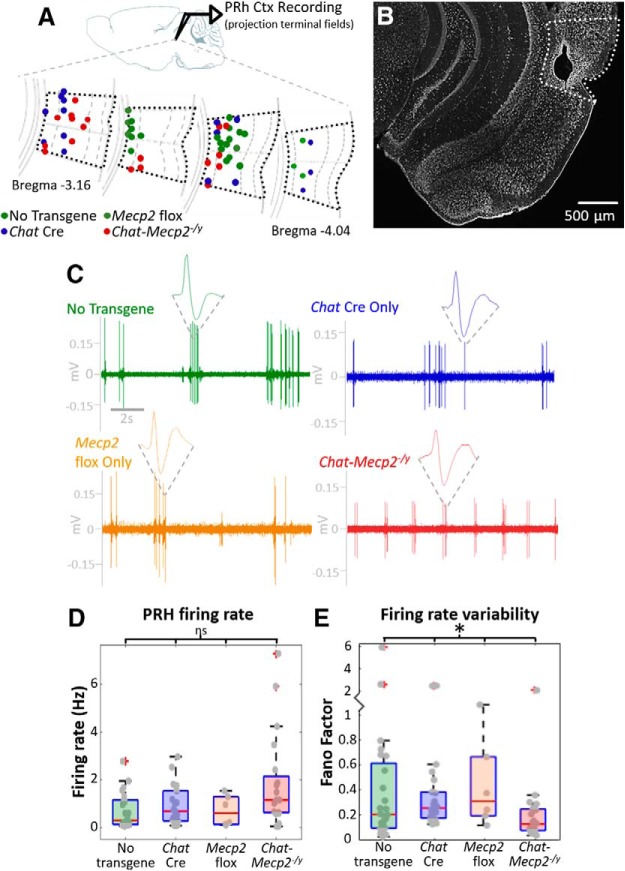
Neuronal firing in the PRH is highly variable, and this variability is lost in *Chat-Mecp2^-/y^* mice. ***A***, *In vivo* recordings were collected from layers 5 and 6 of the PRH. ***B***, Sample Nissl staining and electrolytic lesion marking recording sites in the PRH (white dotted line). ***C***, Representative recordings show the highly variable baseline firing in controls that is lost in *Chat-Mecp2^-/y^* mice. ***D***, There was no difference between genotypes in baseline firing rates in the PRH (Kruskal–Wallis test: *H*_(3)_ = 6.62; *p =* 0.085). ***E***, Variability of firing rate as measured by the Fano factor was significantly different between groups (Kruskal–Wallis test: *H*_(3)_ = 8.92; *p =* 0.03). *Chat-Mecp2^-/y^* mice had a lower firing rate variability than all three controls (no transgene: mean FF = 0.612 ± 1.250; *Chat* Cre: mean FF = 0.474 ± 0.650; *Mecp2* flox: mean FF = 0.447 ± 0.365; *Chat-Mecp2^-/y^*: mean FF = 0.248 ± 0.431). No transgene: *n* = 24 units from 6 mice; *Chat* Cre: *n* = 22 units from 11 mice; *Mecp2* flox: *n* = 6 units from 3 mice; *Chat-Mecp2^-/y^*: *n* = 20 units from 8 mice. *p ≤ 0.05, ns = non significant.

*In vivo* extracellular recordings were collected from L5/6 of the posterior portion of the PRH (Fig. 3*A–C*). Although there was no significant difference in baseline firing rate (Fig. 3*D*; no transgene: *n* = 24 units from 6 mice; *Chat* Cre: *n* = 22 units from 11 mice; *Mecp2* flox: *n* = 6 units from 3 mice; *Chat-Mecp2^-/y^*: *n* = 20 units from 8 mice; Kruskal–Wallis test, *H*_(3)_ = 6.62, *p =* 0.085), PRH units from control mice (WT, *Chat* Cre, and *Mecp2* flox mice) had highly variable firing patterns. In contrast, PRH units from *Chat-Mecp2^-/y^* mice exhibited very regular and rhythmic firing (Fig. 3*C*). Comparison of the variability of the firing rate as measured by the Fano factor revealed a significant difference between groups (Kruskal–Wallis test, *H*_(3)_ = 8.92, *p =* 0.03). PRH units from *Chat-Mecp2^-/y^* mice had the lowest firing rate variability (Fig. 3*E*; no transgene, mean FF = 0.612 ± 1.250; *Chat* Cre, mean FF = 0.474 ± 0.650; *Mecp2* flox, mean FF = 0.447 ± 0.365; *Chat-Mecp2^-/y^*, mean FF = 0.248 ± 0.431).

### Optogenetic stimulation of cholinergic input to the PRH

**Figure 4.**
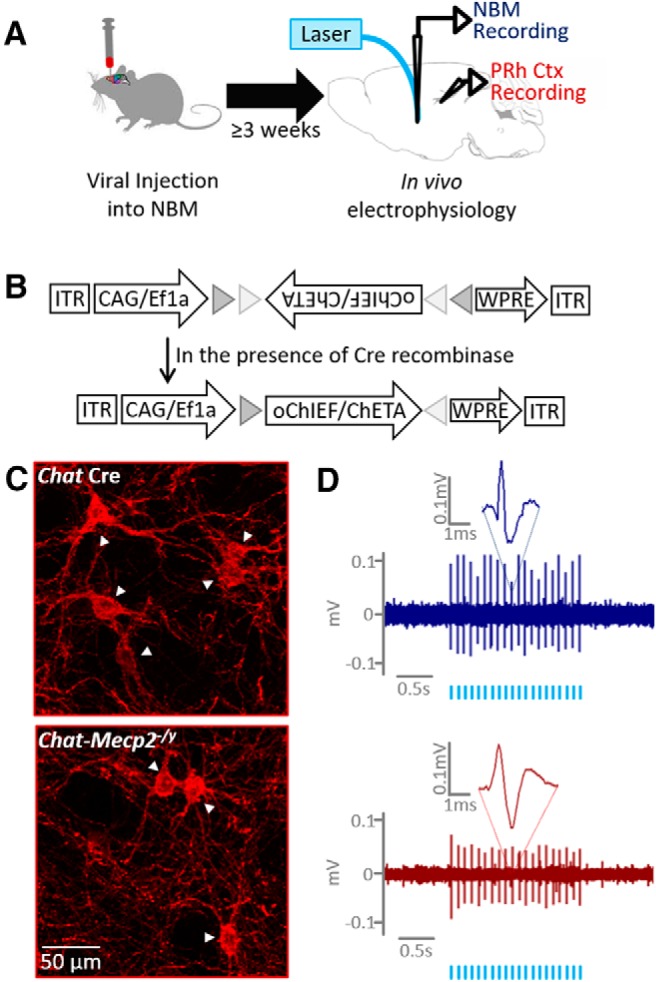
Strategy for optogenetic stimulation of cholinergic neurons. ***A***, Schematic of the experimental paradigm. ***B***, A viral vector encoding an optically activated excitatory ion channel is injected into the NBM. The viral vector is of a flip excision switch design such that it will be expressed only in the presence of Cre recombinase. ***C***, Representative images of virally labeled cholinergic neurons (white arrowheads) from a control mouse (blue, top) and a *Chat-Mecp2^-/y^* mouse (red, bottom). ***D***, Representative optically evoked action potentials in the NBM of a control mouse (top) and a *Chat-Mecp2^-/y^* mouse (bottom). The timing of laser pulses delivered into the NBM is indicated by light blue hash marks.

The above results indicate that *Mecp2* deletion from cholinergic neurons has an important functional effect on PRH firing at baseline. We next asked whether *Mecp2* deletion from cholinergic neurons affected the response of the PRH to the stimulation of endogenous acetylcholine release. The PRH receives the majority of its cholinergic innervation from neurons in the NBM (Woolf, 1991; Kondo and Zaborszky, 2016). To acutely stimulate acetylcholine release in the PRH, we infected *Chat* Cre and *Chat-Mecp2^-/y^* mice with a Cre-dependent AAV (adeno-associated virus) expressing the channelrhodopsin variants oChIEF or ChETA fused to tdTomato (Fig. 4*A*,*B*). Cholinergic neurons in the NBM of both *Chat* Cre and *Chat-Mecp2^-/y^* mice expressed functional oChIEF, as indicated by fluorescent imaging of tdTomato (Fig. 4*C*) and by optically evoked action potentials (Fig. 4*D*).

**Figure 5.**
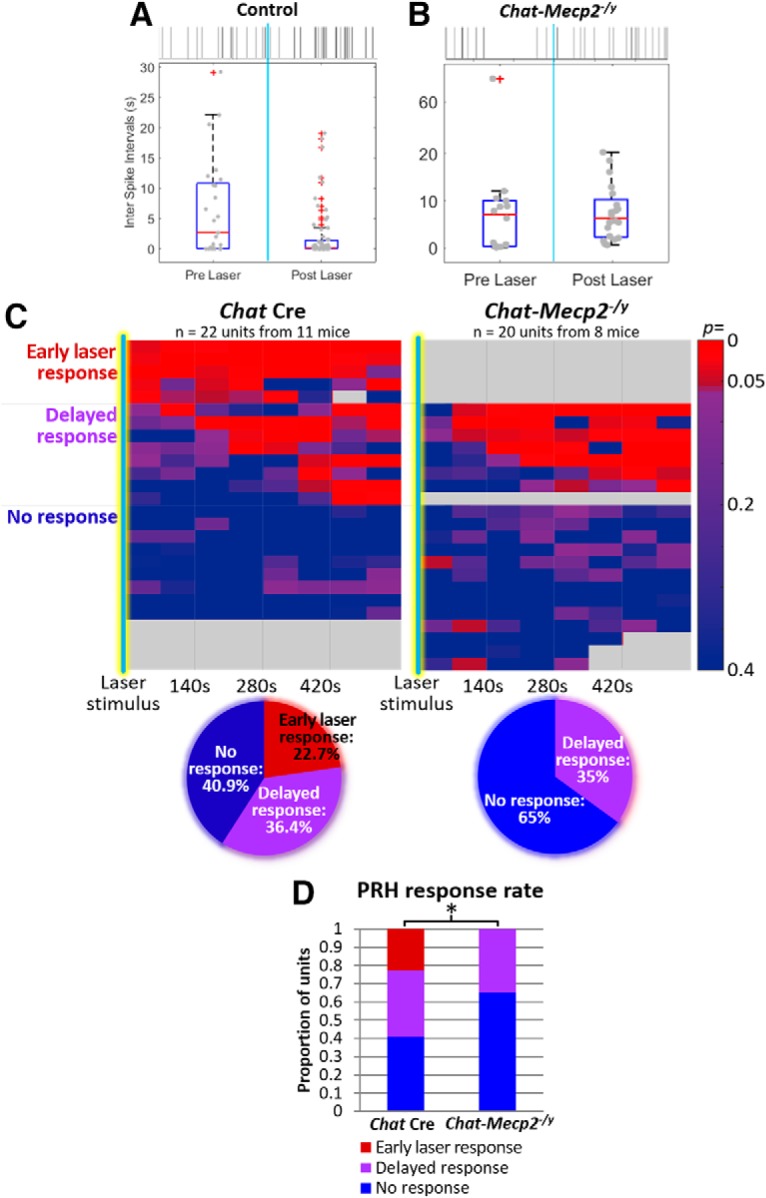
PRH response to the stimulation of endogenous cholinergic signaling is impaired in *Chat-Mecp2^-/y^* mice. ***A***, ***B***, Representative data from a PRH unit in a control mouse exhibiting a response to stimulation of cholinergic input (***A***) and a PRH unit from an *Chat-Mecp2^-/y^* mouse (***B***). Top, Representative raster plot of spikes. Vertical light blue bar indicates timing of optical stimulation. Bottom, Box plot of interspike intervals. ***C***, Heat map of *p* values as a function of time since optical stimulation for PRH units from control mice (left) and from *Chat-Mecp2^-/y^* mice (right). Responses either occurred in the first time period following laser stimulation or were delayed. Each row represents a separate unit. The results are summarized in pie charts at bottom. ***D***, Summary of differing response rates between control and *Chat-Mecp2^-/y^* units (χ^2^ test for homogeneity: CR(2) = 6.02; *p* = 0.049). *p ≤ 0.05.

We next recorded PRH units before, during, and after laser activation of oChIEF expressed in NBM cholinergic neurons (20× 1 ms pulses at 10 Hz). In control animals, the stimulation of NBM cholinergic neurons changed the variance of the interspike intervals (Fig. 5*A*) as measured by the *F* statistic (variance_pre_/variance_post_). PRH units in *Chat-Mecp2^-/y^* did not respond to optical stimulation of cholinergic input; there was no change in ISI variance (Fig. 5*B*) before versus after optical stimulation of cholinergic input.

**Figure 6.**
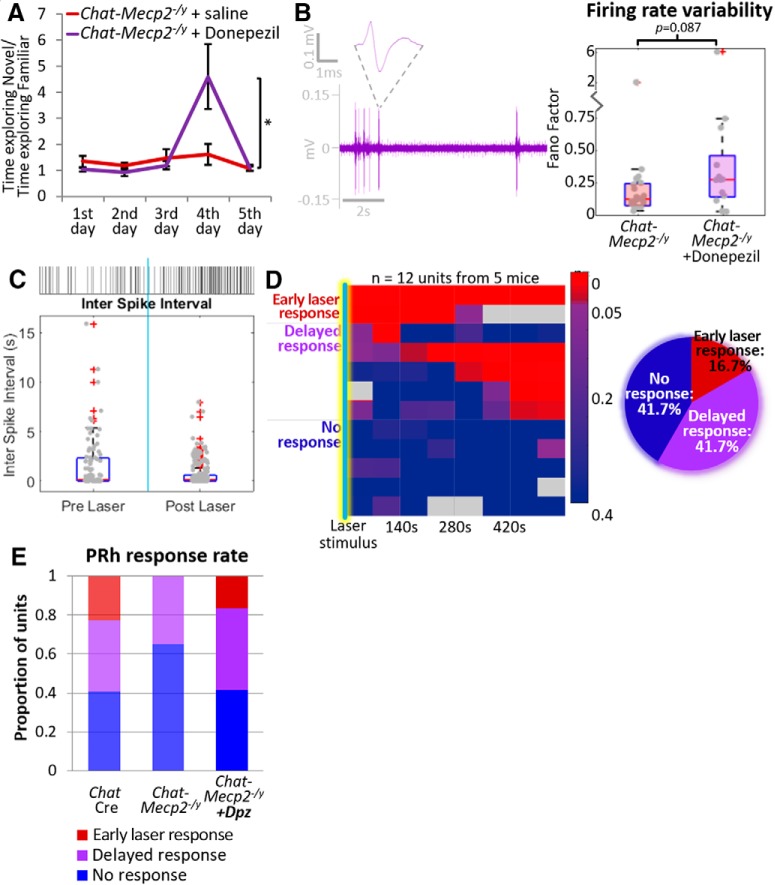
Donepezil treatment of *Chat-Mecp2^-/y^* mice rescues both behavioral and electrophysiological impairments. ***A***, Chronic treatment with systemic donepezil, a drug that inhibits acetylcholinesterase, administered subcutaneously for 2 weeks rescued behavioral impairment (*Chat-Mecp2^-/y^*+ saline, *n* = 8; *Chat-Mecp2^-/y^* + Dpz, *n* = 10; Wilcoxon rank sum test: rank sum = 120; *p* = 0.03). ***B***, Representative raw data trace showing that baseline firing variability was rescued on treatment with donepezil. Firing rate variability was increased in *Chat-Mecp2^-/y^* mice after treatment with donepezil (*Chat-Mecp2^-/y^*, mean FF = 0.248 ± 0.431; *Chat-Mecp2^-/y^*+ Dpz, mean FF = 0.735 ± 1.620), although this difference did not reach statistical significance (Wilcoxon rank sum test: rank sum = 293, *p* = 0.087). ***C***, Sample response to optogenetic stimulation in a *Chat-Mecp2^-/y^* mouse after treatment with donepezil. At top is shown a raster plot of action potentials before and after stimulation of cholinergic input (indicated by light blue vertical bar). At bottom is shown a box plot of interspike intervals obtained before and after optical stimulation of cholinergic neurons in the NBM. ***D***, Heat map of *p* values as a function of time since optical stimulation. Responses either occurred in the first time period following laser stimulation or were delayed and are summarized in the pie chart at bottom. ***E***, Summary of response types. Early laser responses were restored in *Chat-Mecp2^-/y^* mice treated with donepezil. (χ^2^ test for homogeneity: CR(2) = 4.15; *p* = 0.126). *p ≤ 0.05.

Figure 5*C* shows a heat map of response for each PRH unit as a function of time before and after optical stimulation of cholinergic input for control mice (Fig. 5*C*, left) and *Chat-Mecp2^-/y^* mice (Fig. 5*C*, right). While 22.7% of control PRH units (5 of 22) exhibited a change in firing rate variability in the first 140 s time period following laser stimulation, there were no immediate laser stimulation-associated responses detected in *Chat-Mecp2^-/y^* PRH units. The proportion of units exhibiting delayed responses to laser stimulation was similar between control and *Chat-Mecp2^-/y^* mice (*Chat* Cre mice: 36.4%, 8 of 22; *Chat-Mecp2^-/y^*: 35%, 7 of 20). In contrast, the proportion of units with no detectable response to optical stimulation was higher in *Chat-Mecp2^-/y^* mice (65%, 13 of 20) than in controls (40.9%, 9 of 22). These distributions of responses were statistically significantly different (Fig. 6*D*; χ^2^ test for homogeneity, CR(2) = 6.02, *p* = 0.049), which is consistent with a general loss of ACh modulation of PRH activity in the *Chat-Mecp2^-^/y* mice.

### Effect of donepezil on *Chat-Mecp2^-/y^* phenotype

*Mecp2* deletion from cholinergic neurons impaired recognition memory performance and altered PRH cell firing at baseline and after the stimulation of cholinergic projection neurons. If these deficits resulted from impaired cholinergic signaling per se, then we predicted that these phenotypes would be reversed by pharmacological inhibition of acetylcholine degradation by acetylcholinesterase (AChE).

Chronic administration of the AChE inhibitor donepezil (0.3 mg/ml/d, delivered via a subcutaneous minipump) rescued the performance of *Chat-Mecp2^-/y^* mice in the novel object recognition task (Fig. 6*A*). *Chat-Mecp2^-/y^* mice treated with donepezil (*Chat-Mecp2^-/y^* + Dpz) spent significantly more time exploring a novel object than a familiar object (Fig. 6*A*; *Chat-Mecp2^-/y^*+ saline mice, *n* = 8; *Chat-Mecp2^-/y^* + Dpz mice, *n* = 10). In fact, their behavior was qualitatively similar to that of control mice, which is consistent with the idea that donepezil blockade of AChE activity was sufficient to rescue the ability of the mice to distinguish between novel and familiar stimuli.

We next tested whether the electrophysiological effects of cholinergic *Mecp2* deletion could similarly be rescued by donepezil treatment. Figure 6*B* (top) shows a representative extracellular record obtained from a PRH L5/6 neuron in an anesthetized *Chat-Mecp2^-/y^* mouse treated with donepezil. Units recorded in *Chat-Mecp2^-/y^* + Dpz mice had highly variable firing patterns reminiscent of the electrophysiological profiles of control mice (Fig. 5*B*, bottom).

We also tested the effect of donepezil treatment on the PRH response to the stimulation of cholinergic input. Figure 6*C* shows a representative PRH unit from a *Chat-Mecp2^-/y^* + Dpz mouse in which the cholinergic input to the PRH has been optically stimulated. The box plot of ISIs (Fig. 6*C*, left) obtained before and after stimulation shows a change in the variance of ISI. Figure 6*D* shows a heat map of responses of *Chat-Mecp2^-/y^* + DPz PRH units as a function of time since laser stimulation. The early laser response rate of *Chat-Mecp2^-/y^* + DPz PRH units is partially rescued when compared with untreated *Chat-Mecp2^-/y^*, increasing from a value of 0 to 16.7% (2 of 12 PRH units). The proportion of units with delayed responses was slightly higher than in control or *Chat-Mecp2^-/y^* mice (*Chat-Mecp2^-/y^* + Dpz: 41.7%, 5 of 12 PRH units), while the proportion of units with no detectable response was similar to the control levels of ∼40% (41.7%, 5 of 12 PRH units). However, the difference in distribution of response types between *Chat-Mecp2^-/y^* and *Chat-Mecp2^-/y^* + Dpz mice did not reach statistical significance (Fig. 6*E*; χ^2^ test for homogeneity, CR(2) = 4.15, *p* = 0.126).

## Discussion

In this study, we assessed the behavioral and electrophysiological consequences of selective *Mecp2* deletion from cholinergic neurons. Mice with *Mecp2* deletion from cholinergic neurons were selectively impaired in assays of recognition memory. Because of the prominent role of the PRH in recognition memory, we focused our electrophysiological analyses on PRH, where we found subtle alterations in baseline firing of L5/6 neurons in mice with selective knockout of *Mecp2* from cholinergic neurons (Dere et al., 2007). Selective deletion of *Mecp2* from cholinergic neurons also ablated responses to the stimulation of cholinergic input to PRH. Finally, we demonstrated that both the behavioral and the electrophysiological profiles of cholinergic *Mecp2* deletion were rescued by inhibiting ACh breakdown with donepezil.

*Chat-Mecp2^-/y^* mice were selectively impaired in recognition memory tasks—both in tests of novel versus familiar object and in novel versus familiar conspecific recognition. This is consistent with the results of the study by Zhang et al. (2016), who showed that *Chat-Mecp2^-/y^* mice were impaired on the recognition of a familiar conspecific, although they did not assay novel object recognition. The selective nature of the observed cognitive deficit is an intriguing result as cholinergic signaling is known to be vital for performance on both spatial and emotional memory tasks, such as the Morris water maze and the cue-conditioned and/or context-conditioned fear assays (McNamara and Skelton, 1993; Gould, 2003; Jiang et al., 2016). The fact that *Chat-Mecp2^-/y^* mice are not impaired on these tasks implies one of two possibilities. First, not all cholinergic neurons are functionally dependent on *Mecp2* expression. Cholinergic neurons that project to brain areas involved in recognition memory may be functionally dependent on *Mecp2* expression and are therefore functionally impaired by its deletion, while cholinergic neurons that project to areas involved in emotional and spatial memory are not. Although originally conceptualized as homogeneous, cholinergic basal forebrain neurons are actually quite diverse in terms of receptor and neurotransmitter expression and exhibit intricate topographical and functional organization that has only recently begun to be appreciated (Allen et al., 2006; Chandler and Waterhouse, 2012; Chandler et al., 2013; Saunders et al., 2015; Zaborszky et al., 2015a,b; Kondo and Zaborszky, 2016). The second possibility is that the loss of MeCP2 partially impairs cholinergic neurons and that different degrees of impairments might produce different phenotypes. Partial impairment impacts novel object recognition, but it might take more severe impairment to impact other learning phenotypes.

The fact that the deletion of *Mecp2* from cholinergic neurons alters PRH firing both at baseline and after the stimulation of cholinergic input suggests that disruption of cholinergic signaling has several distinct effects on excitability over differing time scales. This is not surprising as ACh is thought to exert its effects via both tonic and transient signaling mechanisms (for review, see Ballinger et al., 2016). The degree to which these different modes of signaling contribute to different or overlapping cognitive functions is not well understood. Our observation that deleting *Mecp2* in cholinergic neurons affects both the baseline rate and pattern of firing of L5/6 PRH neurons as well as the response to stimulation of cholinergic input supports the idea that cholinergic transmission via both of these signaling mechanisms may be involved in the synaptic regulation of PRH neuron excitability.

At baseline, the effect of impairing cholinergic signaling via *Mecp2* deletion is to reduce the variability of firing. This may represent a loss of dynamic range over which individual neurons can encode. The function of ongoing, tonic ACh release in the PRH may therefore be to increase this dynamic range. In addition, the effect of *Chat-Mecp2^-/y^* on the response of PRH neurons to optogenetic stimulation of cholinergic input is to ablate any changes in firing of the target PRH neurons. This may represent reduced functional connectivity of the NBM–PRH circuit. This loss of functional connectivity and dynamic range may impair novel object recognition encoding and therefore underlie the behavioral impairment. As our recordings have all been collected from anesthetized mice, a critical next step is to record from awake animals during behavior performance to clarify the relationship between these electrophysiological phenotypes and behavioral impairment. Furthermore, the current experiments have used cholinergic cell body stimulation to investigate the integrity of the NBM–PRH circuit. However, cholinergic NBM neurons are known to project broadly to a variety of brain areas (see Wu et al., 2014), and it is therefore impossible to determine whether the effects shown here are specific to the PRH or are downstream effects from other cholinergically innervated areas. In fact, when *Mecp2* is re-expressed in cholinergic neurons in *Mecp2* knock-out mice, the deficit in recognition of a familiar conspecific persists, suggesting that, although the present results implicate cholinergic *Mecp2* expression as necessary for intact recognition memory, it is not sufficient (Zhou et al., 2017). Thus, there are likely other relevant brain areas through which the cholinergic basal forebrain exerts its effect on recognition memory performance. Future analysis of the functional connectivity of other brain areas in the context of cholinergic *Mecp2* deletion may clarify their contribution to the behavioral phenotype documented here. Further work investigating cholinergic input to the PRH in *Chat-Mecp2^-/y^* mice using terminal field optogenetic stimulation and neurotransmitter release measurements is also needed to clarify the role of cholinergic signaling in this brain area and the effects of *Mecp2* disruption on it.

It is possible that the electrophysiological and behavioral deficits discussed above are a consequence of a “sick neuron” syndrome induced by the catastrophic effects of *Mecp2* deletion in a subpopulation of neurons or that these effects are mediated by a neurotransmitter other than ACh, as cholinergic neurons are known to synthesize many different neurotransmitters (Tkatch et al., 1998; Allen et al., 2006; Saunders et al., 2015). If these deficits are truly due to a cholinergic signaling impairment, we would expect that they might be rescued by the inhibition of ACh breakdown and boosting of the cholinergic signal. This was indeed the case: the fact that the inhibition of ACh breakdown with donepezil rescued these impairments demonstrates an essential role of ACh per se in mediating the phenotype. The donepezil rescue of behavior was seemingly more robust than the donepezil rescue of the electrophysiological phenotype; however, it is difficult to equate magnitudes across such very different methods.

In conclusion, *Mecp2* deletion from cholinergic neurons leads to selective impairment of recognition memory, reduced variability of PRH firing, and reduced PRH responsivity to the stimulation of cholinergic input. Cholinergic signaling is therefore an important mediator of cognitive deficits in mice lacking MeCP2, and *Mecp2* expression is vital for cholinergic mediation of recognition memory.

## Synthesis

Reviewing Editor: Orly Reiner, Weizmann Institute of Science Decisions are customarily a result of the Reviewing Editor and the peer reviewers coming together and discussing their recommendations until a consensus is reached. When revisions are invited, a fact-based synthesis statement explaining their decision and outlining what is needed to prepare a revision will be listed below.

The reviewers indicated some changes that need to be addressed.

Reviewer 1:

This paper evaluated the role of the cholinergic system to the Rett syndrome phenotype by selectively ablating Mecp2 from cholinergic neurons. Thus, male mice expressing Cre recombinase under the Chat promoter were crossed with female mice carrying a floxed Mecp2 allele. The F1 male offspring was then tested for cognitive performances as well as electrophysiological responses in the posterior perirhinal cortex (PRH). It was found that deletion of Mecp2 specifically in cholinergic neurons disrupted object recognition as well as the recognition of a familiar conspecific, but had no effect on other memory functions such as spatial memory or fear conditioning. Furthermore, at the electrophysiological level, ablation of Mecp2 in cholinergic neurons affected basal firing in the PRH as well as responses to optogenetic stimulation of the NBM that sends projections to the PRH.

General comments:

Rett syndrome is almost predominantly present in females since it is X-linked, so it would have been interesting to take this into account and thus to have focused on female mice or even to have made a sex comparison.

In addition, it would have been interesting to have recorded in a different cortical region than the PRH to determine whether the observed effects are really specific to object recognition and the PRH. For example, it would have been interesting to have analyzed the septo-hippocampal pathway since spatial abilities did not seem to be affected.

Methods:

It is not clear which mouse models were used regarding the optogenetic experiments and viral injections. It is stated Chat Cre mice {plus minus} Mepc2 flox, what does it mean? Were they crossed together and then the F1 generation was used as for the behavior? Same remark for the electrophysiological recordings and the donepezil pump implantation, it is not well explained in the text. What are Mecp2 sKO mice? The Chat-Cre crossed with floxed Mecp2? The nomenclature has to be more consistent throughout the ms.

Furthermore, did all mice undergo the same battery of tests or were there subgroups each time (the n's seem to differ between the behavioral tests), so it seems unlikely but some explanations on which mice were used for which experiments are needed.

Results: it is abundant to mention the results in the text, the figure legends and in the figures themselves. It affects the legibility of the ms in general.

Regarding the optogenetic experiments, it is not clear what “laser-locked response” means functionally speaking. Furthermore, the authors conclude that optogenetic stimulation of the NBM eliminated responses of PRH neurons in Chat-Mecp2-/y. Figure 6A shows indeed no changes in interspike intervals but does this necessarily mean that they do not respond? Amplitudes could have been changed for instance. Overall, these data are not easy to follow and in particularly their functional meaning.

Minor comments:

Figure 2E, the Y-axis, please add that it is the ratio between the time exploring the novel vs the familiar object.

Page 8, Table 1 should be Table 2.

Figure 6D is not mentioned in the text.

Figure 7A: why not have included a control group of Chat Cre mice?

Reviewer 2:

In this interesting study, the authors demonstrated that mice with Mecp2 deletion from cholinergic neurons are selectively impaired in recognition memory, which is mediated by the perirhinal cortex (PRH). Deletion of Mecp2 from cholinergic neurons resulted in profound alterations in baseline firing of L5/6 pyramidal neurons and eliminated responses of these neurons to optogenetic stimulation of cholinergic input to PRH. Both the behavioral and the electrophysiological deficits of cholinergic Mecp2 deletion are rescued by inhibiting ACh breakdown with donepezil treatment.

The authors investigated the behavioral deficits in Chat-Mecp2-/y mice at circuit level by using optogenetic approach and in vivo electrophysiological recording. While the findings are interesting, several concerns should be addressed.

General comments:

1. The writing of manuscript need to be improved. For example, the title of the manuscript did not match with the content. It is better to show your interested brain region, PRH in the title. In the introduction section, there is no any information related with PRH. The different distributions of responses in Figure 6C indicated ...... (page9)? In significance statement section, the authors did not incorporate all the findings in this manuscript, such as therapeutic effect by donepezil.

2. The authors showed several behavioral tests in Figure 2. In human studies, the gray matter reduction of basal forebrain is found in ASD children. The projection regions of cholinergic neurons from the basal forebrain include mPFC and hippocampus, excepting perirhinal cortex (PRH), where involve in social behavior, communication, and cognitive skills. In Figure2F, the results showed that Chat-Mecp2-/y mice spent similar time at partition as other strains and increased time with reintroduced familiar mice compared to others. The authors wrote “These data are consistent with the idea that selective deletion of Mecp2 from cholinergic neurons alters the animals' ability to distinguish between novel and familiar stimuli”. Importantly, the ability to discriminate between familiar and novel individuals is not impaired in Chat-Mecp2-/y mice. The increased time spent with reintroduced mice in Chat-Mecp2-/y mice indicated social novelty preference or social memory may be impaired in Chat-Mecp2-/y mice. But it is hard to say by using the method in this study. Three-chamber sociability and social novelty preference, and Two-trial (or five-trial) social memory test may clarify it. Social ability is mainly associated with PFC and/or hippocampus. The authors can re-emphasize this result since this paper focused on PRH.

3. For electrophysiological experiments, the authors claimed the extracellular recording cells are pyramidal cells in PRH. There are interneurons in PRH. There is no any interpretation to distinguish pyramidal cells and interneurons in manuscript by EP or staining. Please explain in discussion. In figure 3B, figure 5D and figure 7B, the different scales of Y-axis were used. The reason may be from different distances between recording cells and electrodes. It is better to use the same scale. Regarding to the representative trace for cholinergic Mecp2 deletion mice in figure 3B, I am concerning the cell type. Please add one representative trace recorded from interneuron in figure 3B. Add two representative traces in figure 7B.

4. The authors improperly used statistical method. For example, in figure 2E and figure 7A, the correct statistical method need to used is repeated two-way ANOVA. There are two variants, one is time variant in both graphs, the other is genetic variant (Figure 2E) or treatment variant (Figure 7A).

5. Explain in detail about Chat-Mecp2-/y mice with reduced survival in method section. Whether female Chat-Mecp2-/y mice showed similar behavioral impairments.

6. The quality of images needs to be improved. In Figure 1, the location of the images, from BF? Scale Bar? Quantification of loss Mecp2 by measurement of the intensity of fluorescence. If Quantification was shown in your precious or others studies, please cite it. What is the electrophysiological consequence of loss of Mecp2 in cholinergic neuron itself? Does it affect cholinergic neuronal excitability (firing frequency)?

7. I am wondering what your Nissl stainings look like, which is used to show the recording sites. Whether a representative image with high magnification can be shown in Figure 3A.

8. Can Figure 4 incorporate into Figure 3? Clarify how many mice used along with the cell numbers in your electrophysiological experiments.

9. Systemic administration of the AChE inhibitor restored the impairment of novel object recognition (Figure 7). I am wondering whether it restored the behavioral deficits in Figure 2F. The electrophysiological results are not as strong as behavioral results. Explain the potential reasons in discussion section.
